# The morphometric features derived as the predictors of acute ischemic stroke recurrence

**DOI:** 10.3389/fnins.2026.1755459

**Published:** 2026-08-27

**Authors:** Jianzheng Sun, Peng Dong, Lijing Wang, Jinfeng Long, Yinuo Qi, Limei Song

**Affiliations:** 1 School of Medical Imaging, Shandong Second Medical University, Weifang, China; 2 School of Clinical Medicine, Shandong Second Medical University, Weifang, China

**Keywords:** acute ischemic stroke, machine learning, cortical morphometry, prediction, recurrence

## Abstract

**Background and objective:**

Several approaches have been used to estimate the risk of recurrent ischemic stroke, including clinical risk scores, models based on routine clinical data, and diffusion-weighted imaging radiomics. Most of these methods, however, focus on established clinical factors or features of the acute infarct and do not fully reflect structural changes across the whole brain. It remains uncertain whether morphometric measures derived from T1-weighted imaging can improve the prediction of recurrence within 1 year. We therefore evaluated the predictive value of these measures.

**Methods:**

A total of 201 subjects with acute ischemic stroke (AIS) were assigned to the recurrence and nonrecurrence groups. Cortical morphometric features were extracted from T1-weighted imaging (T1WI) using FreeSurfer. Three feature selection strategies were evaluated: independent-samples t-tests alone, least absolute shrinkage and selection operator (LASSO) regression alone, and independent-samples t-tests followed by LASSO regression. Eight machine learning models were developed using the selected features, resulting in 24 feature selection model combinations. Model performance was evaluated using the area under the receiver operating characteristic curve (AUC), accuracy, sensitivity, specificity, precision, F1 score, calibration analysis, and decision curve analysis. Shapley additive explanations (SHAP) were used to assess feature importance and interpret model predictions.

**Results:**

Among the 24 feature selection model combinations, SVM using features selected by independent-samples t-tests followed by LASSO regression achieved the highest AUC, with an AUC of 0.841 (95% CI, 0.786–0.897), accuracy of 0.771, sensitivity of 0.705, specificity of 0.824, precision of 0.792, and F1 score of 0.738. Calibration analysis showed that this model had the lowest Brier score of 0.162, while decision curve analysis demonstrated a favorable net benefit across a broad range of threshold probabilities. SHAP analysis identified the folding index of the left medial orbital–olfactory sulcus and the mean curvature of the left subcallosal gyrus as important contributors to model predictions.

**Conclusion:**

This interpretable and reproducible model is a valuable tool for predicting ischemic stroke recurrence, aiding clinical risk stratification and informing individualized treatment decisions.

## 1 Introduction

Acute ischemic stroke (AIS) is a leading cause of global mortality and long-term disability, imposing a substantial social and economic burden (Feigin and Owolabi, 2023). Although considerable progress has been made in acute treatment and prevention, the rates of stroke recurrence remain unacceptably high. Approximately 1 in 8 patients experienced a recurrence stroke within the first year, with many facing a persistently elevated risk thereafter (Amarenco et al., 2018; Flach et al., 2020; Xu et al., 2022). Additional neurological deficits after a recurrent stroke may increase patients vulnerability to secondary complications such as pneumonia, urinary tract infections, and sepsis, ultimately leading to poorer clinical outcomes (Skajaa et al., 2022). Therefore, accurately identifying individuals at high risk of recurrence is crucial for tailoring effective management and improving clinical outcomes.

Current approaches to stroke recurrence prediction include clinical and imaging risk scores, as well as machine learning models based on routine clinical data or radiomic features extracted from acute infarcts (Ay et al., 2010; Arsava et al., 2016; Wang et al., 2022; Liu et al., 2023; Li et al., 2024). Although these approaches capture established vascular risk factors, stroke mechanisms, and lesion properties, lesion-centered characteristics alone may not fully reflect the distributed effects of stroke on the brain. Focal ischemic injury can induce structural alterations in remote but anatomically connected regions, a process commonly attributed to diaschisis and secondary degeneration. These alterations include degeneration of connecting white matter pathways, focal cortical thinning, and remote gray-matter atrophy (Duering et al., 2012; Cheng et al., 2015; Duering et al., 2015; Wei et al., 2019; Cheng et al., 2020; Geng et al., 2023). Quantitative assessment of cortical morphology may therefore provide complementary information for recurrence risk evaluation.

Diffusion-weighted imaging (DWI) is widely used for AIS diagnosis because it can detect ischemic changes within minutes of symptom onset. However, DWI provides limited anatomical detail and is less suitable for characterizing fine cortical morphology, including cortical thickness, surface area, and gyral-sulcal architecture. T1-weighted imaging (T1WI) combined with FreeSurfer enables automated quantification of regional cortical thickness, surface area, cortical gray matter volume, curvature, and folding (Fischl, 2012). Previous studies have linked cortical morphometric measures to functional recovery, post-stroke cognitive impairment, and structural brain health (Rojas Albert et al., 2022; Liew et al., 2023). One study used FreeSurfer-derived cortical thickness together with clinical and imaging variables to predict major neurocognitive disorder 3 months after stroke, with an SVM model achieving an AUC of 0.876; regional cortical thickness measures in the left occipital and temporal cortices and right cingulate cortex contributed to model prediction (Aamodt et al., 2021). However, these studies primarily focused on functional or cognitive outcomes, examined cortical thickness alone, or summarized multiple measures into a global index. Whether multiple regional cortical morphometric measures can contribute to AIS recurrence prediction remains unclear.

The present study evaluated whether T1WI-derived cortical morphometric measures could be used to predict AIS recurrence within 1 year. Specifically, we aimed to: (1) extract cortical thickness, surface area, cortical gray matter volume, curvature, and folding measures using FreeSurfer; (2) identify candidate cortical morphometric features associated with 1-year AIS recurrence through statistical and machine learning-based feature selection; and (3) develop and internally evaluate multiple machine learning models for individual-level recurrence prediction. Feature-importance analyses were further performed to identify the cortical measures contributing most strongly to model predictions.

## 2 Materials and methods

Cortical morphometric features were extracted from T1WI using FreeSurfer, followed by data preprocessing and quality control. Three feature selection strategies were evaluated: independent-samples t-tests alone, LASSO regression alone, and independent-samples t-tests followed by LASSO regression. Features selected using each strategy were used to train eight machine learning models: logistic regression (LR), support vector machine (SVM), multilayer perceptron (MLP), k-nearest neighbors (KNN), decision tree (DT), gradient boosting decision tree (GBDT), linear discriminant analysis (LDA), and adaptive boosting (AdaBoost). Model performance was evaluated using the area under the receiver operating characteristic curve (AUC), accuracy, sensitivity, specificity, precision, F1 score, calibration analysis, and decision curve analysis (DCA).

### 2.1 Data acquisition

**Figure 1:**
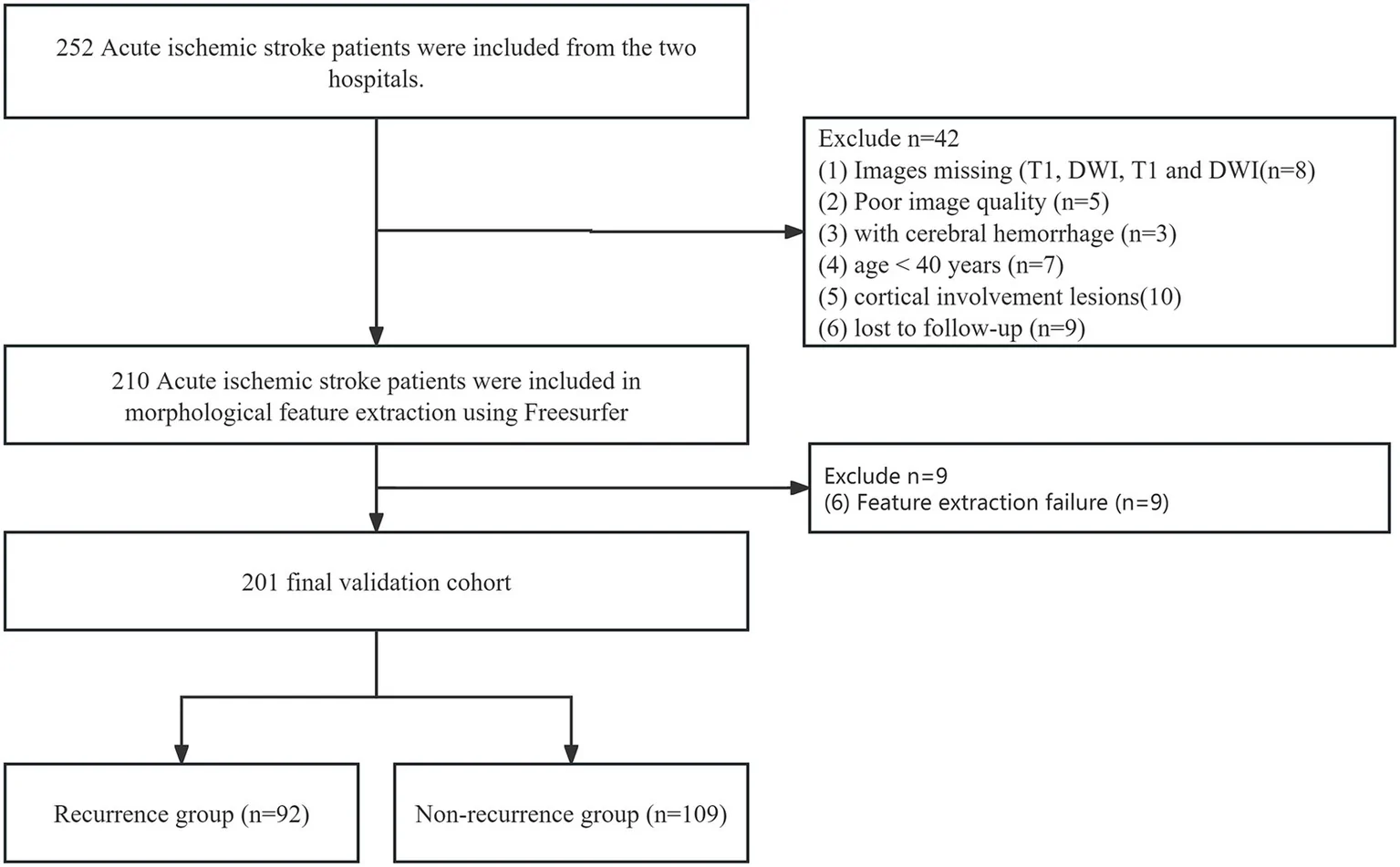
The flowchart of the analyzed study population.

From 2013 to 2022, a total of 201 subjects were enrolled from the Department of Neurology of Affiliated Hospital of Shandong Second Medical University. All subjects were followed up for 1 year to monitor whether the subjects experienced AIS recurrence. As shown in Figure 1, the cohort was categorized into two groups: the recurrence group (92 subjects, including 51 males and 41 females) and the nonrecurrence group (109 subjects, 63 males and 46 females).

The study inclusion criteria were: (1) AIS diagnosed by both clinical symptoms and imaging findings and (2) onset within 72 h. Exclusion criteria included the following reasons: (1) age < 40; (2) pre-existing neurological or psychiatric disorders, cerebral tumors, cerebral hemorrhage, history of substance abuse, malignant cerebral edema, lesions involving the cerebral cortex; (3) Subjects were excluded for severe MRI artifacts, images missing, poor image quality, mortality during follow-up or loss to follow-up.

MRI scans were acquired using a 1.5 T Achieva Philips superconducting scanner equipped with an 8-channel SENSE parallel acquisition head coil at Affiliated Hospital of Shandong Second Medical University. Axial DWI was performed using a single-shot echo-planar imaging sequence with the following parameters: TR/TE = 3000/75 ms, slice thickness = 6 mm, interslice gap = 1.5 mm, and b-values = 0 and 1,000 s/mm^2^. Spin-echo T1WI was acquired with TR/TE = 500/20 ms, slice thickness = 6 mm, and interslice gap = 1.5 mm. All subjects were instructed to remain still during scanning to minimize the motion artifacts.

### 2.2 Overview of the proposed architecture for predicting AIS recurrence

**Figure 2:**
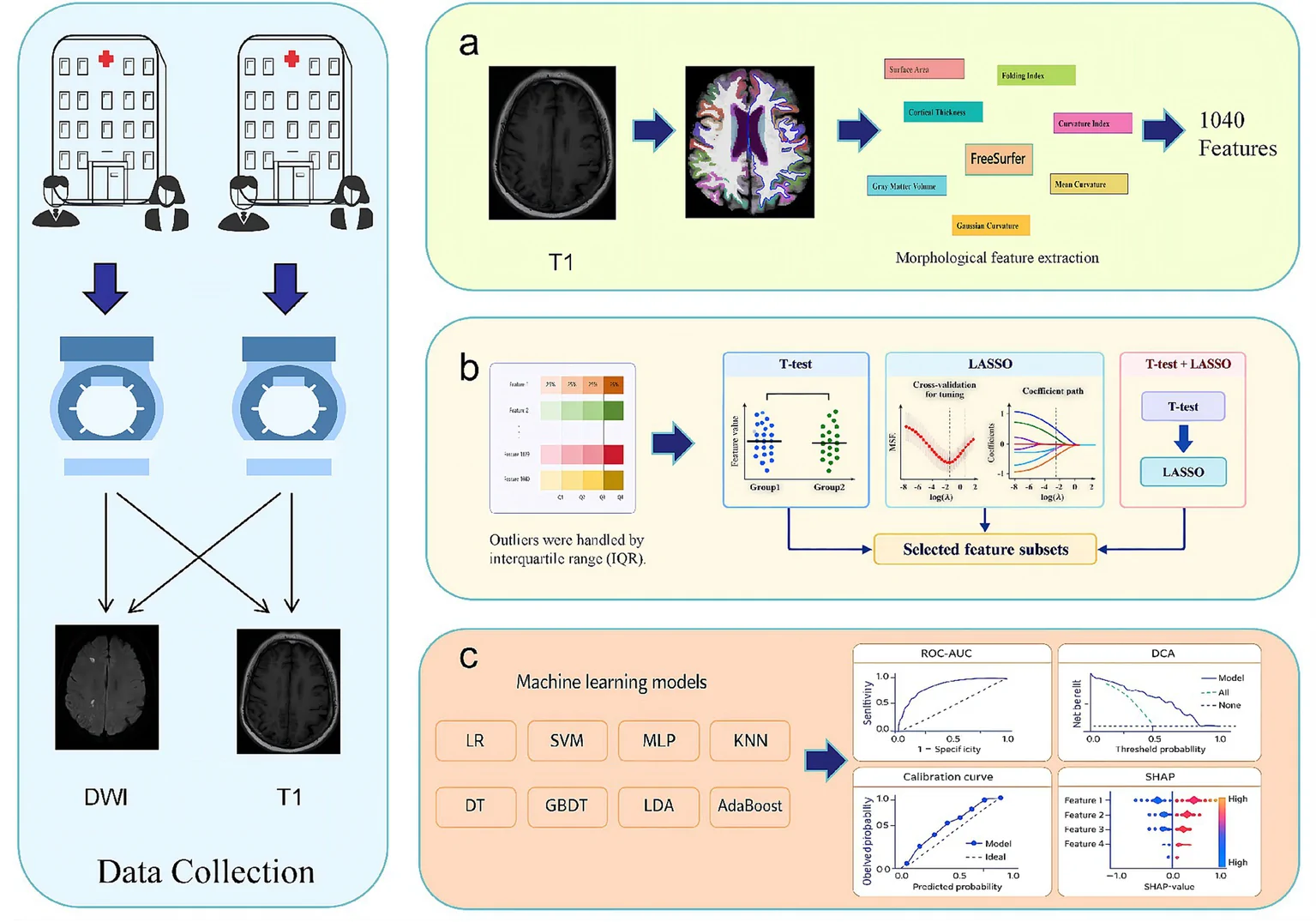
Overview of the proposed framework for AIS recurrence prediction. **(a)** Extraction of cortical morphometric features from T1WI using FreeSurfer; **(b)** Feature selection using independent-samples *t*-tests, LASSO regression, and independent-samples *t*-tests followed by LASSO regression; and **(c)** development and evaluation of eight machine learning models.

Figure 2 illustrates the overall workflow of the present study for predicting AIS recurrence: (1) cortical morphometric features were extracted from each subject using FreeSurfer; (2) three feature selection strategies, including independent-samples t-tests, LASSO regression, and independent-samples t-tests followed by LASSO regression, were applied for dimensionality reduction; and (3) the selected cortical morphometric features were used to develop machine learning models for predicting AIS recurrence.

### 2.3 Preprocessing and morphometric feature extraction

A full processing stream for structural T1WI data was applied using FreeSurfer^1^, encompassing intensity correction, skull stripping, surface co-registration, spatial smoothing, segmentation of white matter and subcortical structures, and cortical parcellation. A total of 1,040 cortical morphometric features were extracted from T1WI images using the recon-all pipeline of FreeSurfer. Specifically, the Destrieux atlas (a2009s) (Destrieux et al., 2010) divides each cerebral hemisphere into 74 cortical regions, yielding 148 cortical regions in total. For each of these regions, seven morphometric measures were extracted: Surface Area, Cortical Thickness, Cortical Gray Matter Volume, Curvature Index, Folding Index, Gaussian Curvature, and Mean Curvature. This resulted in 148 regions × 7 measures = 1,036 features for each individual. Additionally, mean white matter surface area and mean cortical thickness of each hemisphere were computed resulting in a total of 1,040 cortical morphometric features. The processed images were manually inspected and edited for accuracy by two experienced radiologists.

### 2.4 Feature selection

The dataset was divided into training and validation sets in a 7:3 ratio using stratified sampling to preserve the proportions of recurrence and nonrecurrence cases. All feature selection procedures were performed exclusively in the training set. Data quality was controlled as follows: (1) any feature with > 10% missing or outlier values was excluded; (2) Missing values for each feature were imputed using feature-wise median; (3) Outliers were identified using the interquartile range (IQR) criterion (values below Q1–1.0 × IQR or above Q3 + 1.0 × IQR) and replaced with the median calculated within the corresponding dataset.

Three feature selection strategies were evaluated: independent-samples t-tests alone, LASSO regression alone, and independent-samples t-tests followed by LASSO regression. For the independent-samples t-test strategy, each cortical morphometric feature was compared between the recurrence and nonrecurrence groups in the training set, and features with *p* < 0.0001 were retained. For the LASSO strategy, features were standardized and entered into an L1-penalized logistic regression model. Features were standardized and entered into an L1-penalized logistic regression model implemented with the liblinear solver. The inverse regularization parameter, C, was optimized over 10 logarithmically spaced values ranging from 10^−4^ to 10^4^ using stratified ten-fold cross-validation based on the minimum cross-validated log loss. The model was subsequently refitted on the complete training set using the optimal C, and features with nonzero coefficients were retained. For the combined strategy, features with *p* < 0.05 were first identified using independent-samples t-tests and subsequently subjected to the same LASSO procedure for further feature selection.

### 2.5 Prediction model

In this study, eight machine learning algorithms, including LR, SVM, MLP, KNN, DT, GBDT, LDA, and AdaBoost, were used to develop binary classification models for predicting AIS recurrence based on imaging features. Grid search combined with five-fold cross-validation was performed within the training set to optimize model hyperparameters and reduce the risk of overfitting. After model development, the final models were evaluated on the independent validation set. Model performance was assessed using accuracy, sensitivity, specificity, precision, F1 score, and AUC. These metrics were reported as point estimates with 95% confidence intervals, which were estimated using 2,000 bootstrap resamples. Calibration analysis was performed to assess the agreement between predicted probabilities and observed outcomes, and DCA was conducted to evaluate the potential clinical utility of the models across a range of threshold probabilities. The hardware configuration used in this study is presented in Supplementary Table S1, and the detailed hyperparameter settings for all models are summarized in Supplementary Table S2.

### 2.6 Statistical analysis

Descriptive statistics for continuous variables were summarized as mean with standard deviation (SD) and were compared using independent-samples t-tests. Categorical variables were compared using the chi-square test. *p* < 0.05 was considered statistically significant. All statistical analyses were conducted in Python (version 3.10.16) using the SciPy library (version 1.15.3).

## 3 Results

### 3.1 Basic characteristics

There were no statistically significant differences in age or sex between the recurrence and nonrecurrence groups (*p* > 0.05) (Table 1).

**Table 1:** The detailed information about the subjects.

Characteristics	Recurrence group (*n* = 92)	Nonrecurrence group (*n* = 109)	*p*
Mean age (*y*)	66.05 ± 9.85 (mean ± SD)	65.05 ± 9.51 (mean ± SD)	>0.05
Sex	51/41 (M/F)	62/47 (M/F)	>0.05

### 3.2 Feature selection and comparative performance of prediction models

**Figure 3:**
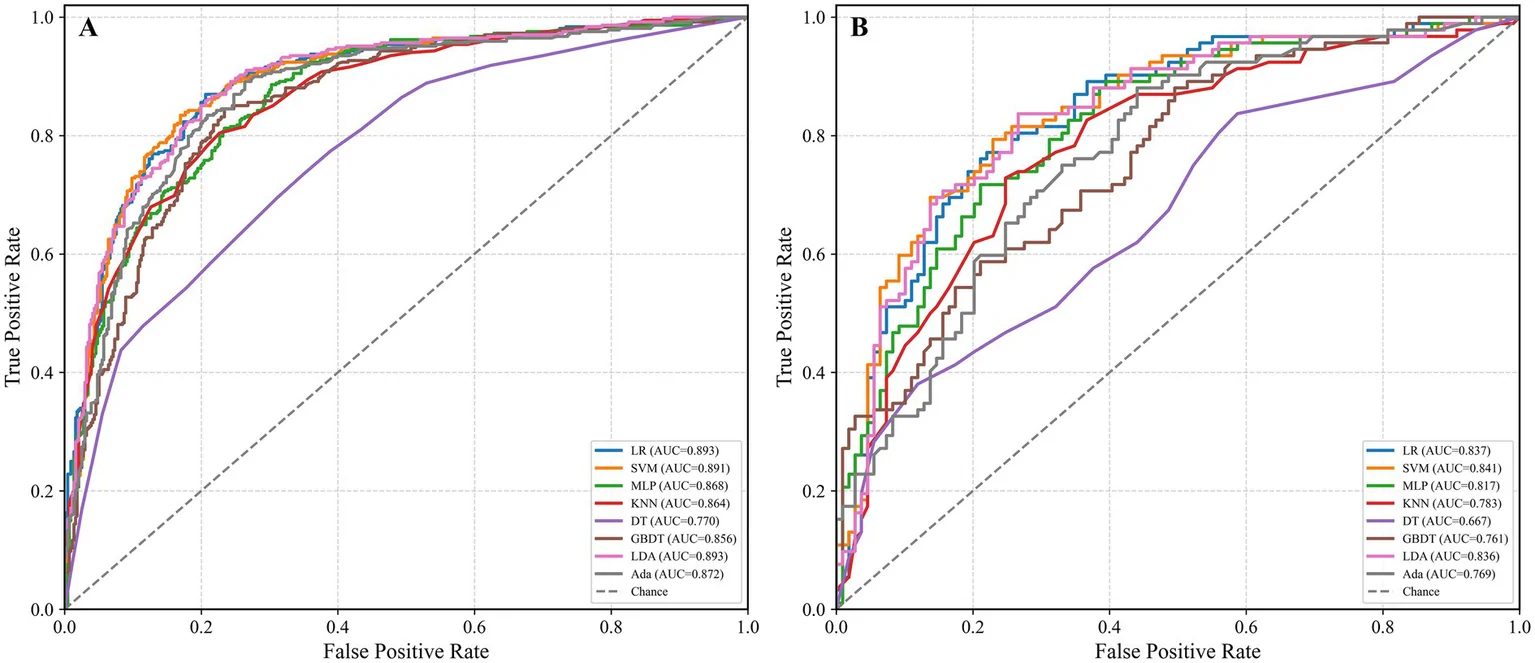
ROC curves of the eight machine learning models based on cortical morphometric features selected by independent-samples *t*-tests followed by LASSO regression: **(A)** training set and **(B)** validation set.

**Figure 4:**
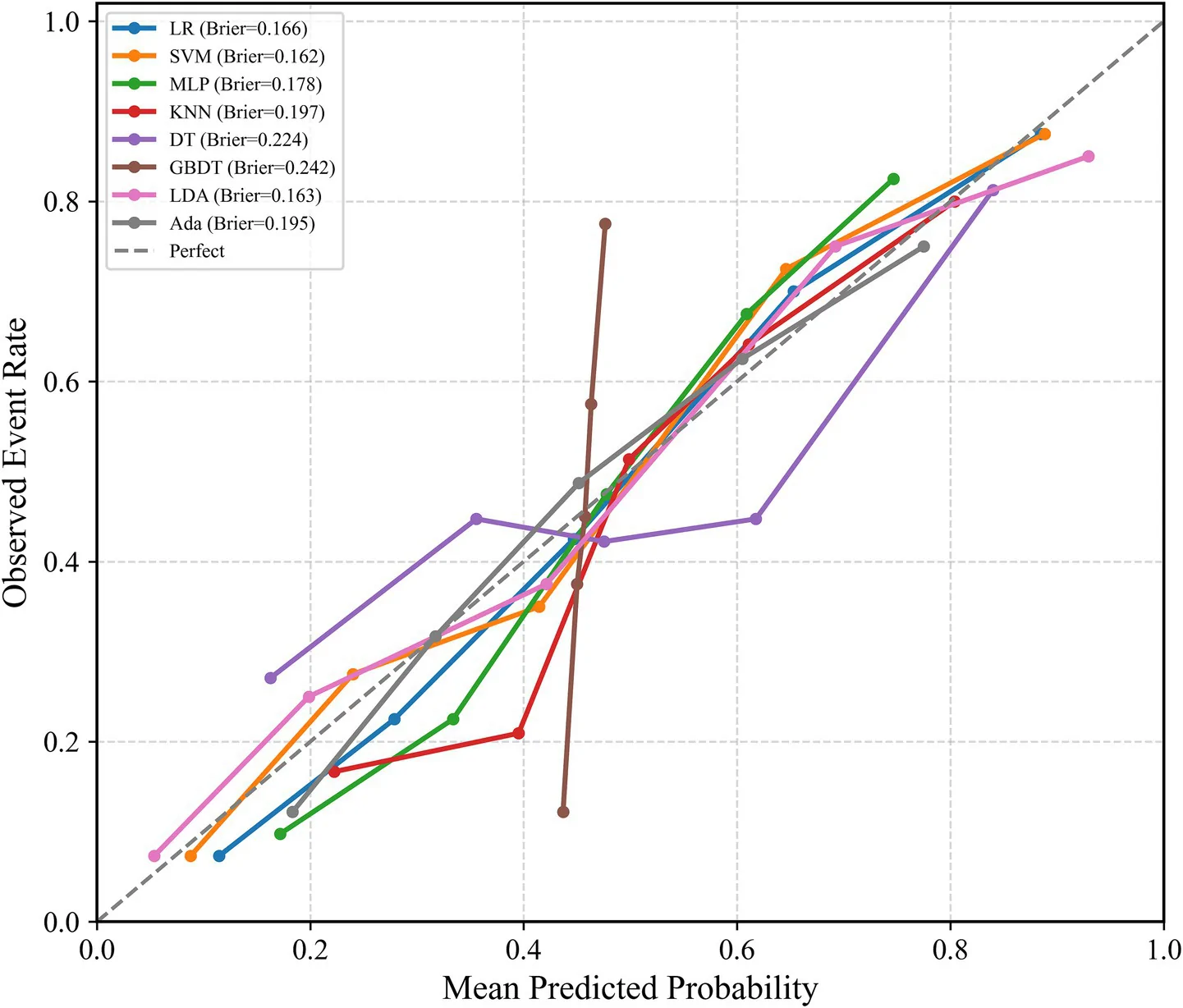
Calibration curves of the eight machine learning models based on cortical morphometric features selected by independent-samples t-tests followed by LASSO regression in the validation set.

**Figure 5:**
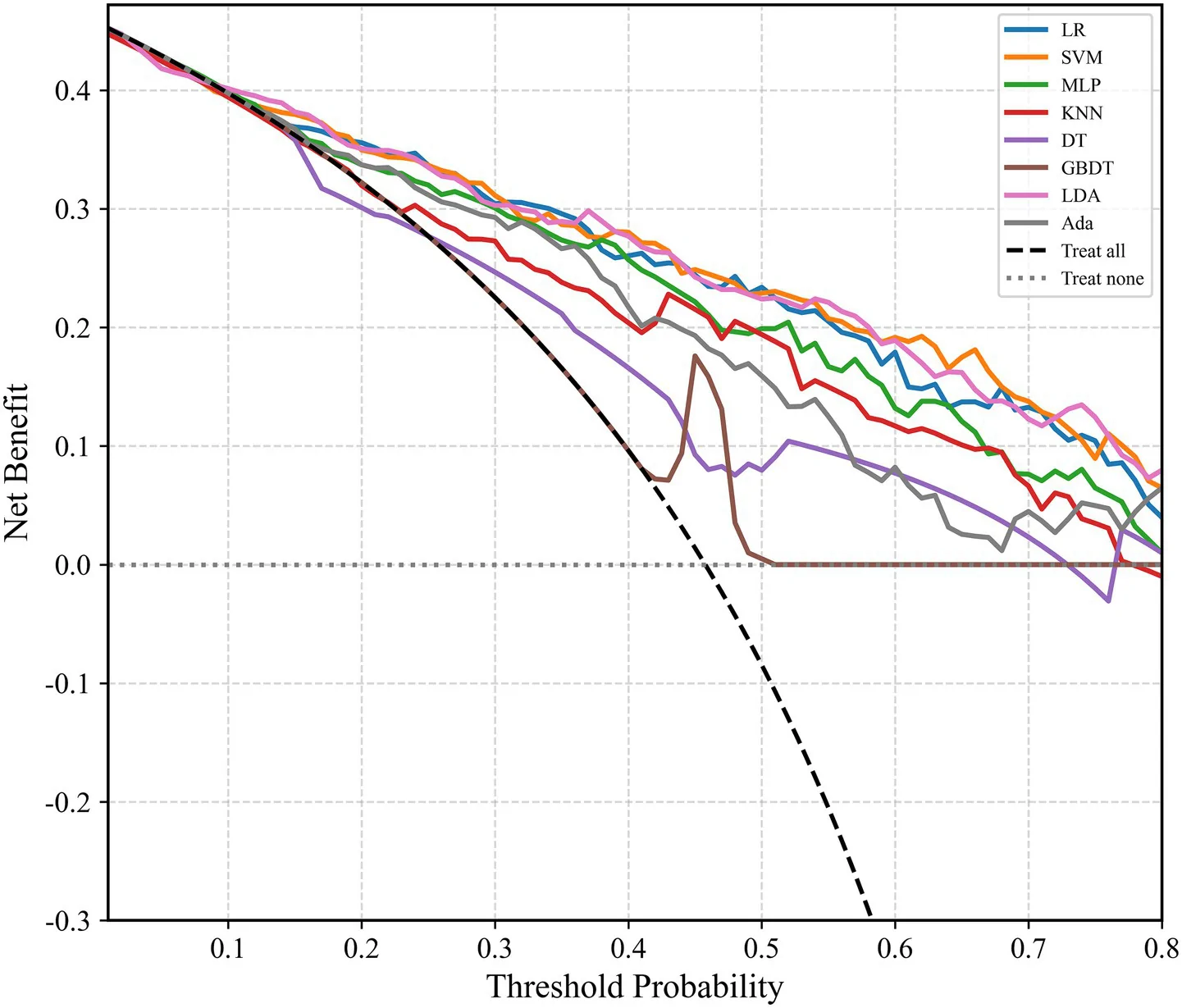
Decision curve analysis of the eight machine learning models based on cortical morphometric features selected by independent-samples t-tests followed by LASSO regression in the validation set.

All feature selection procedures were performed exclusively in the training set. Independent-samples t-tests, LASSO regression, and independent-samples t-tests followed by LASSO regression retained 12, 7, and 13 features, respectively, which were applied unchanged to the validation set. Across the eight machine learning models, the corresponding average AUCs were 0.734, 0.747, and 0.789, respectively, with the combined strategy showing the highest overall performance (Table 2). Among all 24 feature selection model combinations, SVM based on features selected by independent-samples t-tests followed by LASSO regression showed relatively good performance, with an accuracy of 0.771, sensitivity of 0.705, specificity of 0.824, precision of 0.792, F1 score of 0.738, and AUC of 0.841 (Table 3; Figure 3). This model also showed the lowest Brier score of 0.162 (Figure 4) and favorable net benefit across a broad range of threshold probabilities (Figure 5).

**Table 2:** AUCs of eight machine learning models under different feature selection strategies.

Feature selection strategies	AUC of machine learning models							
	LR	SVM	MLP	KNN	DT	GBDT	LDA	AdaBoost
Independent-samples t-tests	0.768	0.767	0.734	0.747	0.615	0.738	0.777	0.727
LASSO regression	0.759	0.776	0.741	0.737	0.711	0.760	0.774	0.718
Independent-samples *t*-tests followed by LASSO regression.	0.837	0.841	0.817	0.783	0.667	0.761	0.836	0.769
Average	0.788	0.795	0.764	0.756	0.664	0.753	0.796	0.738

**Table 3:** Performance of eight machine learning models based on features selected using independent-samples t-tests followed by LASSO regression.

Model	Accuracy	Sensitivity	Specificity	Precision	F1 score	AUC
LR	0.776 (0.735–0.817)	0.749 (0.689–0.808)	0.797 (0.691–0.902)	0.772 (0.688–0.856)	0.755 (0.720–0.789)	0.837 (0.768–0.904)
SVM	0.771 (0.734–0.808)	0.705 (0.634–0.775)	0.824 (0.714–0.933)	0.792 (0.709–0.874)	0.738 (0.712–0.764)	0.841 (0.786–0.897)
MLP	0.741 (0.692–0.789)	0.728 (0.675–0.779)	0.752 (0.690–0.814)	0.715 (0.654–0.776)	0.720 (0.669–0.772)	0.817 (0.761–0.872)
KNN	0.711 (0.652–0.771)	0.696 (0.604–0.787)	0.725 (0.637–0.806)	0.681 (0.590–0.774)	0.688 (0.612–0.760)	0.783 (0.719–0.844)
DT	0.622 (0.552–0.687)	0.467 (0.370–0.570)	0.752 (0.670–0.827)	0.614 (0.500–0.722)	0.531 (0.434–0.616)	0.667 (0.588–0.740)
GBDT	0.652 (0.582–0.716)	0.652 (0.551–0.747)	0.651 (0.558–0.738)	0.612 (0.515–0.708)	0.632 (0.547–0.708)	0.761 (0.694–0.823)
LDA	0.746 (0.687–0.806)	0.707 (0.615–0.798)	0.780 (0.704–0.856)	0.730 (0.636–0.823)	0.718 (0.639–0.788)	0.836 (0.780–0.890)
AdaBoost	0.682 (0.617–0.741)	0.674 (0.576–0.767)	0.688 (0.596–0.775)	0.646 (0.545–0.744)	0.660 (0.573–0.733)	0.769 (0.701–0.831)

Data are presented as point estimates with 95% confidence intervals (CIs).

### 3.3 Interpretable results of prediction models

**Figure 6:**
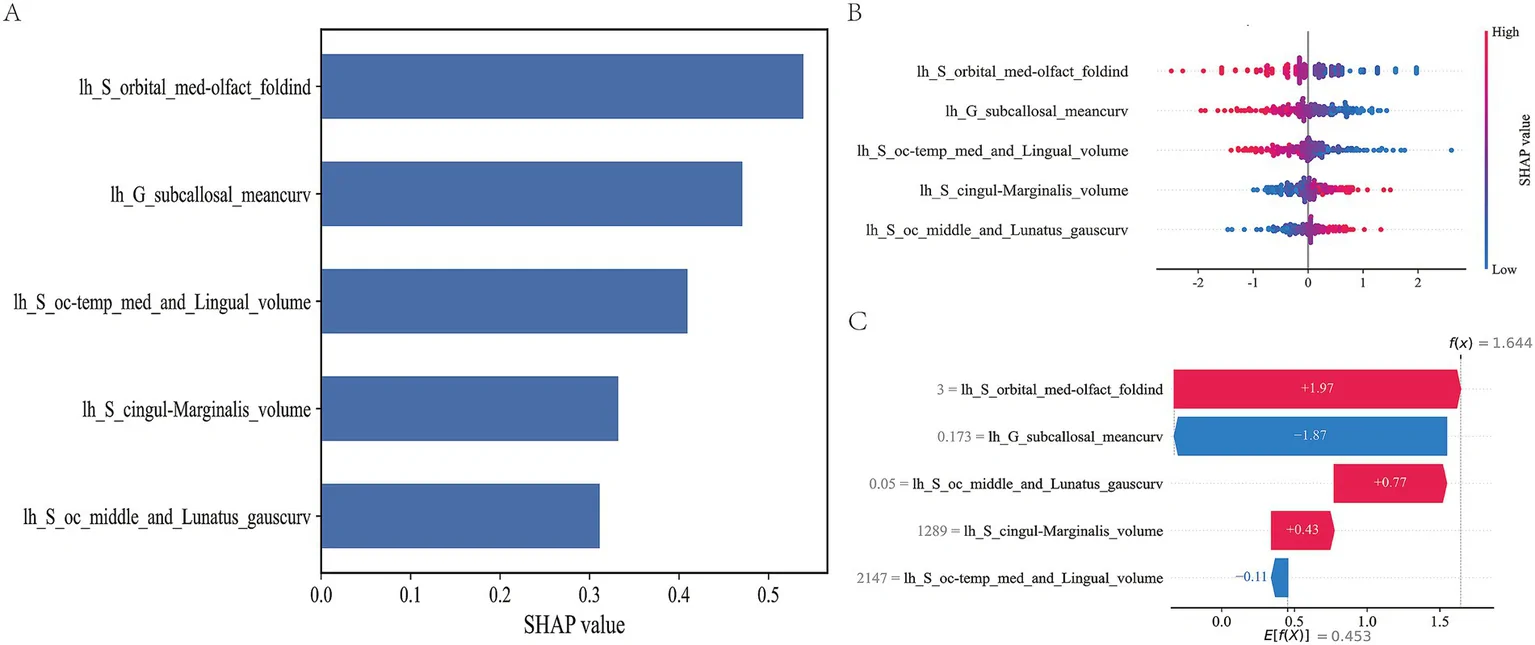
SHAP visualizations for the Support Vector Machine model. Top five features ranked by absolute SHAP value. **(A)** SHAP values illustrating the relative importance of the top cortical features contributing to the model’s prediction of recurrence risk **(B)** beeswarm plot demonstrating the distribution of SHAP values for each feature across all subjects, with color gradients reflecting corresponding feature magnitudes **(C)** waterfall plot for an individual case, detailing the directional influence of each feature on the deviation of the predicted risk from the model’s baseline output.

Figure 6A displays the SHAP-based feature importance of the SVM model developed for AIS recurrence prediction. Each bar represents the mean absolute SHAP value of a feature, reflecting its overall importance to the model predictions. Typically, a higher mean absolute SHAP value indicated greater feature importance in the model’s predictions. SHAP analysis of the SVM model indicated that a folding index feature (lh_S_orbital_med-olfact_foldind) and a mean curvature feature (lh_G_subcallosal_meancurv) were among the most influential predictors of AIS recurrence (see Supplementary Table S3 for abbreviations and definitions).

The beeswarm plot illustrates how variation in each cortical feature influences the model output across the cohort. Each point represents a subject, with the color indicating the feature value (pink for higher values and blue for lower values), and the horizontal position representing the SHAP value, showing whether that feature pushes the prediction upward or downward (Figure 6B).

Positive contributions are shown in red and increase the model output relative to the base value, while negative contributions are shown in blue and decrease the output. Each bar represents the magnitude of a feature contribution to the model output. For this subject, the predicted value was *f*(*x*) = 1.644, driven mainly by two cortical measures. lh_S_orbital_med-olfact_foldind produced the strongest positive shift (+1.97), indicating a substantial increase in the predicted recurrence risk. In contrast, lh_G_subcallosal_meancurv showed a notable negative contribution (−1.87), reflecting an effect in the opposite direction. The waterfall plot demonstrates how these major feature-specific influences combine to form the individualized risk estimate (Figure 6C).

## 4 Discussion

The present study developed a machine learning based framework utilizing cortical morphometric features extracted from T1WI images to predict the 1-year recurrence risk in AIS subjects. In this framework, spatially distributed variations in cortical morphology that are not readily appreciable on routine MRI review are quantified as model input features, and the resulting models demonstrate good discriminatory performance in distinguishing subjects. These findings provide valuable insights for identifying individuals at high risk of recurrence, thereby facilitating personalized management and improving clinical outcomes.

### 4.1 Key findings

A majority of neuroimaging-based prediction studies (Silva et al., 2017; Chen et al., 2023) have focused on morphometric measures such as cortical thickness, surface area, and cortical gray matter volume to forecast neurological and psychiatric outcomes. Building upon these conventional morphometric measures, the present study further integrates curvature and folding-related features (curvind, foldind, gauscurv, and meancurv), providing a more comprehensive representation of cortical geometry and structural complexity. These geometric descriptors reflect the intrinsic structural complexity of the brain and have been associated with both neurodevelopmental patterns and pathological remodeling (Demirci and Holland, 2022), potentially capturing subtle cortical alterations that extend beyond thickness- or volume-based measures.

In this study, 1,040 cortical morphometric features were extracted using the Destrieux atlas and evaluated using three feature selection strategies and eight machine learning models. Among all combinations, SVM with features selected by independent-samples t-tests followed by LASSO regression showed relatively good performance, with an AUC of 0.841. The combined feature selection strategy may help identify informative cortical features for AIS recurrence prediction. Notably, this model showed higher specificity than sensitivity, indicating a greater ability to correctly identify subjects without recurrence, although some subjects who subsequently experienced recurrence were not correctly identified. Overall, these findings support the potential value of combining cortical morphometric features with machine learning for AIS recurrence prediction and individualized risk assessment.

### 4.2 SHAP identified orbitofrontal and subcallosal contributors to AIS recurrence

SHAP was applied to enhance model interpretability and clarify feature contributions. In the SVM model, the two features with the highest SHAP values were lh_S_orbital_med-olfact_foldind and lh_G_subcallosal_meancurv, indicating their dominant roles in predicting AIS recurrence (Figure 6). The feature with the highest mean absolute SHAP value in SVM model was lh_S_orbital_med-olfact_foldind a region within the orbitofrontal cortex (OFC) that has reveived relatively limited research attention. This region plays critical roles in emotion regulation, decision-making, and reward processing (Rolls et al., 2020). Neuropsychiatric symptoms including depression, irritability, eating disturbances, agitation, apathy and anxiety have been identified in post-stroke populations (Angelelli et al., 2004). Structural or functional alterations in the OFC has been linked to these post-stroke neuropsychiatric symptoms(Carrarini et al., 2021; Ahn et al., 2022; Chu et al., 2024; Tang et al., 2024; Gruschow et al., 2025). Although several studies have revealed the OFC alterations in subjects with ischemic stroke, its potential role in AIS recurrence remains to be elucidated.

The second most important feature identified by SHAP analysis was lh_G_subcallosal_meancurv. According to the Destrieux atlas, G_subcallosal comprises both the Subcallosal area and the subcallosal gyrus (Destrieux et al., 2010). Although the subcallosal gyrus as a whole remains relatively understudied, one of its components, the Subcallosal Cingulate (SCC), has been extensively investigated (Dunlop et al., 2017; Tsolaki et al., 2021; Sobstyl et al., 2022). Aberrant metabolic activity in the SCC of subjects with major depressive disorder was observed by Hamani et al. (2011), and this abnormality was reversible after antidepressant treatment. Previous research has also demonstrated that post-stroke depression is associated with an increased risk of recurrent cerebrovascular events (Wu et al., 2019). Our study revealed that the top two morphological features with the highest predictive contributions were located in brain regions involved in emotional and behavioral regulation (Mayberg, 2009; Rolls, 2023). These findings align with the present results, indicating that affective disturbances after stroke may contribute to an unfavorable long-term. Cerebrovascular prognosis.

### 4.3 Clinical implications

Several studies have highlighted the value of neuroimaging features in understanding stroke risk and in aiding the personalized medical treatmens (Włodarczyk et al., 2022; Gaviria and Eltayeb Hamid, 2024). In this study, a novel prediction model to bridge cortical morphometric features with the risk of AIS recurrence was developed. Current assessment of the risk of AIS recurrence relies predominantly on clinical factors and traditional risk scoring systems which offer limited predictive power and fail to consider individual brain structural variations. The new prediction approach in this study capturing the cortical morphometric information that clinical evaluations alone may miss enhances the predictive accuracy. Although this model cannot replace clinical evaluations, it serves as a complementary decision-support tool. By leveraging machine learning to analyze cortical morphology, it aids clinicians in identifying high-risk subjects for closer monitoring and in tailoring personalized prevention and treatment strategies.

### 4.4 Limitations

Several limitations should be considered. Firstly, the cortical morphometric features were extracted from T1WI data acquired from a single scanner model at a single institution. The applicability of our findings to other imaging settings or diverse populations may be restricted. Although internal validation and multiple algorithms were employed to enhance model robustness, external validation in independent, multi-center cohorts is essential to confirm the model’s reproducibility. Secondly, clinical variables were not incorporated into the predictive model. Integrating multifaceted clinical data, including genetic, biomarker, clinical history, lifestyle factors and MRI imaging features can establish a more comprehensive prediction framework to improve the accuracy of AIS recurrence prediction. This meaningful prediction model can support more personalized AIS recurrence information in monitoring and tailoring personalized prevention and treatment strategies.

## 5 Conclusion

By pioneering the integration of cortical morphometry and machine learning, this study establishes a reproducible framework for predicting AIS recurrence. Using cortical morphometric features derived from T1WI data,the model identified cortical morphometric features that contributed to AIS recurrence prediction. These findings confirm the feasibility of this morphometry-based approach and underscore its potential to yield interpretable, clinically actionable insights for personalized patient management.

## Data Availability

The datasets presented in this article are not readily available because the data that support the findings of this study are available on request from the corresponding author. Requests to access the datasets should be directed to LS, songlm@sdsmu.edu.cn.

## Footnotes

1
https://surfer.nmr.mgh.harvard.edu/
