# Supplementary material for: The morphometric features derived as the predictors of acute ischemic stroke recurrence
Source: Front Neurosci. 2026 Aug 27;20:1755459. doi: 10.3389/fnins.2026.1755459 (PMC13558140; doi:10.3389/fnins.2026.1755459)
Supplement: Supplementary file 1 [file Table_1.DOCX]

Supplementary Material

**Table S1.** Detailed hardware configuration list.

| Operating system | Ubuntu 20.04.6 LTS |
| --- | --- |
| GPU | GeForce RTX 2080 Ti GPU × 1 |
| CPU | Intel(R) Xeon(R) Silver 4216 CPU @ 2.10GHz |
| Memory | 125 GB |

Table S2. Hyperparameter Settings for Eight Machine Learning Models

| Parameter | Logistic Regression | Support Vector Machine | Multi-Layer Perceptron | K-Nearest Neighbors | Decision Tree | Gradient Boosting Decision Tree | Linear Discriminant Analysis | AdaBoost |
| --- | --- | --- | --- | --- | --- | --- | --- | --- |
| C | 0.1, 0.02, 0.05 | 0.01, 0.1, 1, 10, 100 | N/A | N/A | N/A | N/A | N/A | N/A |
| Penalty | l2 (default = l2) | N/A (linear kernel only) | N/A | N/A | N/A | N/A | N/A | N/A |
| Solver | liblinear (default = lbfgs) | N/A | N/A | N/A | N/A | N/A | svd, lsqr | N/A |
| Class weight | {0:1, 1:1} | 'balanced' | N/A | N/A | None, balanced | N/A | N/A | balanced (base estimator) |
| Hidden layer sizes | N/A | N/A | (10,), (20,) | N/A | N/A | N/A | N/A | N/A |
| Activation function | N/A | N/A | relu, tanh | N/A | N/A | N/A | N/A | N/A |
| Alpha (L2 penalty) | N/A | N/A | 0.01, 0.1 | N/A | N/A | N/A | N/A | N/A |
| Max iterations | 100 | N/A | 100 | N/A | N/A | N/A | N/A | N/A |
| Number of neighbors | N/A | N/A | N/A | 7, 9, 11, 13, 15, 17, 19, 21 | N/A | N/A | N/A | N/A |
| Weights | N/A | N/A | N/A | uniform | N/A | N/A | N/A | N/A |
| Minkowski parameter (p) | N/A | N/A | N/A | 1, 2 | N/A | N/A | N/A | N/A |
| Metric | N/A | N/A | N/A | minkowski | N/A | N/A | N/A | N/A |
| Max depth | N/A | N/A | N/A | N/A | 1, 2 | 1 | N/A | 1 (base estimator) |
| Min samples split | N/A | N/A | N/A | N/A | 30, 50 | 30, 40 | N/A | 20 (base estimator) |
| Min samples leaf | N/A | N/A | N/A | N/A | 15, 25, 35 | 15, 25 | N/A | 10 (base estimator) |
| Max leaf nodes | N/A | N/A | N/A | N/A | 2, 3, 4 | N/A | N/A | N/A |
| Max features | N/A | N/A | N/A | N/A | sqrt, 0.5 | sqrt | N/A | sqrt (base estimator) |
| ccp_alpha | N/A | N/A | N/A | N/A | 0.01, 0.03, 0.05, 0.10 | 0.01, 0.03 | N/A | N/A |
| Number of estimators | N/A | N/A | N/A | N/A | N/A | 10, 20, 30 | N/A | 10, 20, 30, 40, 50 |
| Learning rate | N/A | N/A | N/A | N/A | N/A | 0.005, 0.01 | N/A | 0.005, 0.01, 0.03, 0.05 |
| Subsample | N/A | N/A | N/A | N/A | N/A | 0.4, 0.6 | N/A | N/A |
| Shrinkage | N/A | N/A | N/A | N/A | N/A | N/A | None, auto, 0.1, 0.5, 0.9 (lsqr only) | N/A |
| Base estimator | N/A | N/A | N/A | N/A | N/A | N/A | N/A | Decision tree |

Table S3. Details of the selected features

| **Long name** | **Feature name** |
| --- | --- |
| Medial orbital sulcus (olfactory sulcus) | lh_S_orbital_med-olfact_foldind |
| Subcallosal area, subcallosal gyrus | lh_G_subcallosal_meancurv |
| Middle occipital sulcus and lunatus sulcus | lh_S_oc_middle_and_Lunatus_gauscurv |
| Marginal branch (or part) of the cingulate sulcus | lh_S_cingul-Marginalis_volume |
| Medial occipito-temporal sulcus (collateral sulcus) and lingual sulcus | lh_S_oc-temp_med_and_Lingual_volume |

**Note:** lh = left hemisphere, rh = right hemisphere.
